# Supplementary figures and images for: The effects of afforestation on soil bacterial communities in temperate grassland are modulated by soil chemical properties
Source: PeerJ. 2019 Jan 11;7:e6147. doi: 10.7717/peerj.6147 (PMC6330960; doi:10.7717/peerj.6147)

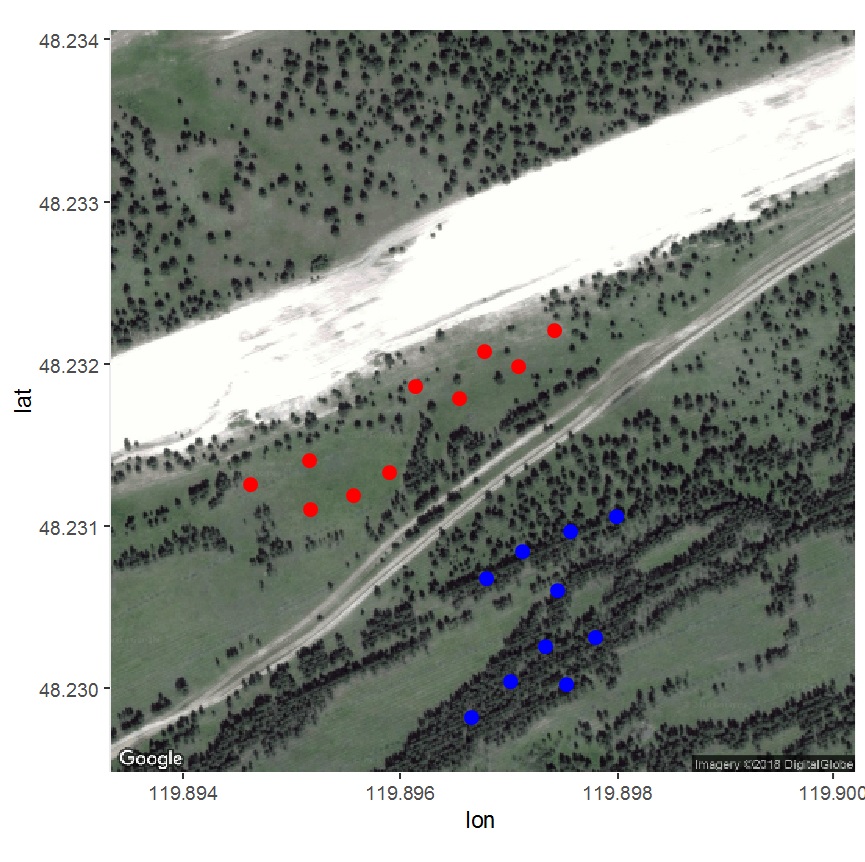

Supplement: Figure S1 — Map data ©2018 Google. [file peerj-07-6147-s001.jpg]

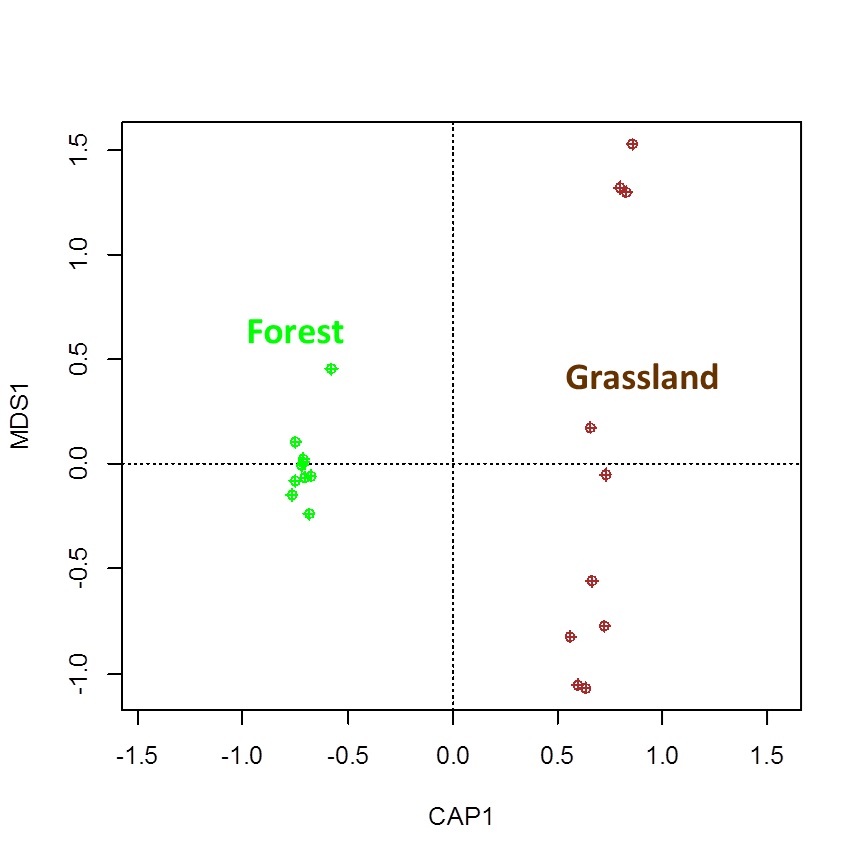

Supplement: Figure S2 [file peerj-07-6147-s002.jpg]

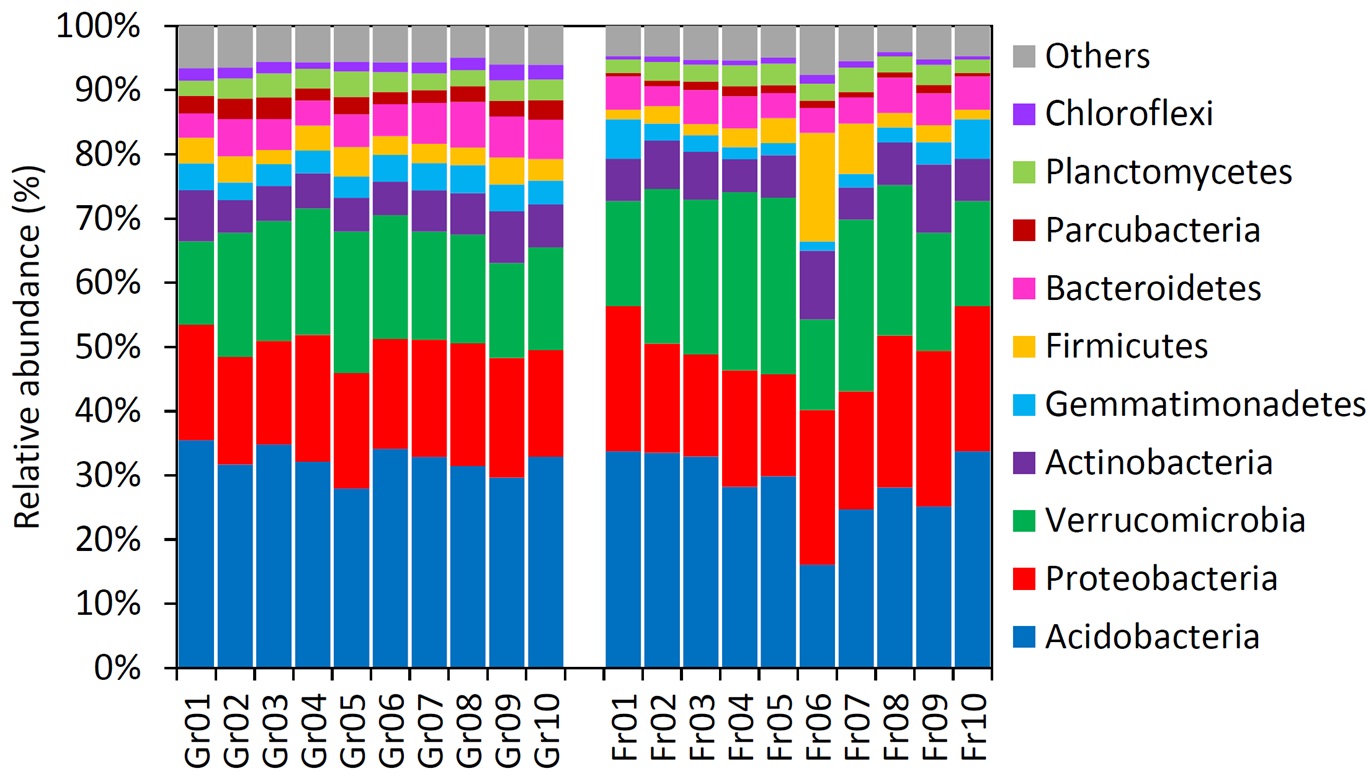

Supplement: Figure S3 [file peerj-07-6147-s003.jpg]

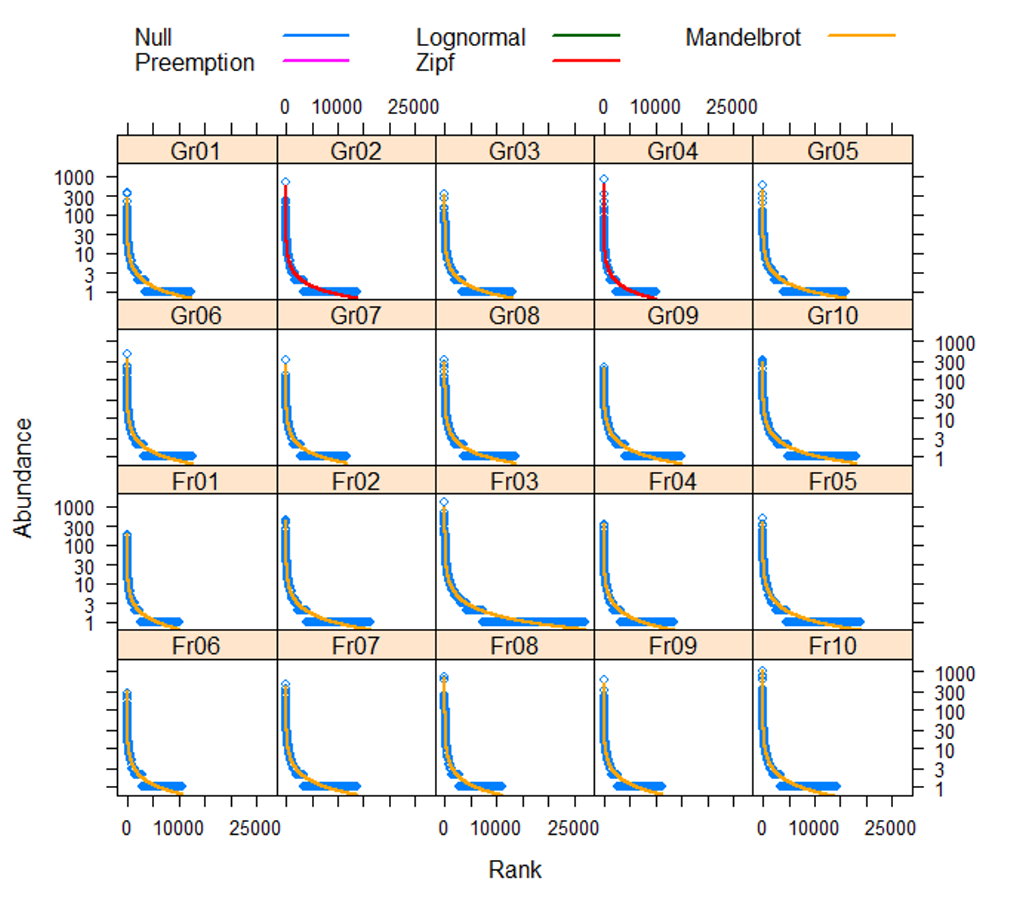

Supplement: Figure S4 — The blue dots and the line in each panel are the observed values and the expectations simulated from the observed data, respectively. Gr and Fr indicate samples from the grassland and forest soils, respectively. The Zipf and Zipf–Mandelbrot models are both niche-based models. Rejection of the null model indicates rejection of the hypothesis of a stochastic process of microbial assembly. [file peerj-07-6147-s004.jpg]
