# Supplementary material for: The effects of afforestation on soil bacterial communities in temperate grassland are modulated by soil chemical properties
Source: PeerJ. 2019 Jan 11;7:e6147. doi: 10.7717/peerj.6147 (PMC6330960; doi:10.7717/peerj.6147)
Supplement: Table S1 — A quasi-Poisson distribution model was used for overdispersed COGs. [file peerj-07-6147-s005.doc]

**Table S1.** Correlations of the COGs and KEGG modules predicted by the soil microbiome with the soil elements P, Ca, and Fe that significantly explain the variance of the soil microbiome from dbRDA by the generalized linear model (GLM). A quasi-Poisson distribution model was used for overdispersed COGs.

|  | P | |  | Ca | |  | Fe | | Distribution model | COG_Description |
| --- | --- | --- | --- | --- | --- | --- | --- | --- | --- | --- |
|  | *z* (or *t*) | *P* |  | *z* (or *t*) | *P* |  | *z* (or *t*) | *P* |  |  |
| **COG Number** |  |  |  |  |  |  |  |  |  |  |
| COG1532 | 4.660 | **3E-04** |  | 3.736 | **0.002** |  | -4.956 | **1E-04** | quasi-Poisson | Predicted RNA-binding protein |
| COG1688 | 3.177 | **0.006** |  | 2.587 | **0.020** |  | -3.539 | **0.003** | quasi-Poisson | Uncharacterized protein predicted to be involved in DNA repair (RAMP superfamily) |
| COG1693 | 5.404 | **6E-05** |  | 5.048 | **1E-04** |  | -5.777 | **3E-05** | quasi-Poisson | Uncharacterized protein conserved in archaea |
| COG1750 | 4.287 | **6E-04** |  | 3.502 | **0.003** |  | -4.712 | **2E-04** | quasi-Poisson | Archaeal serine proteases |
| COG1754 | -4.472 | **4E-04** |  | -4.822 | **2E-04** |  | 5.920 | **2E-05** | quasi-Poisson | Uncharacterized C-terminal domain of topoisomerase IA |
| COG1895 | 4.781 | **2E-04** |  | 4.023 | **1E-03** |  | -5.211 | **9E-05** | quasi-Poisson | Uncharacterized conserved protein related to C-terminal domain of eukaryotic chaperone, SACSIN |
| COG1906 | 4.606 | **3E-04** |  | 3.662 | **0.002** |  | -4.940 | **1E-04** | quasi-Poisson | Uncharacterized conserved protein |
| COG2254 | -4.300 | **2E-05** |  | -4.782 | **2E-06** |  | 5.512 | **4E-08** | Poisson | Predicted HD superfamily hydrolase, possibly a nuclease |
| COG2413 | -3.626 | **3E-04** |  | -3.175 | **0.002** |  | 4.609 | **4E-06** | Poisson | Predicted nucleotidyltransferase |
| COG2946 | 5.281 | **7E-05** |  | 4.954 | **1E-04** |  | -5.701 | **3E-05** | quasi-Poisson | Putative phage replication protein RstA |
| COG3018 | 4.840 | **2E-04** |  | 4.075 | **9E-04** |  | -5.181 | **9E-05** | quasi-Poisson | Uncharacterized protein conserved in bacteria |
| COG3192 | -0.425 | 0.676 |  | 1.231 | 0.236 |  | 0.409 | 0.688 | quasi-Poisson | Ethanolamine utilization protein |
| COG3374 | 3.034 | **0.008** |  | 2.622 | **2E-02** |  | -3.456 | **0.003** | quasi-Poisson | Predicted membrane protein |
| COG3543 | 2.547 | **0.022** |  | 1.853 | 0.082 |  | -3.012 | **0.008** | quasi-Poisson | Uncharacterized conserved protein |
| COG3630 | -0.441 | 0.665 |  | 1.301 | 0.212 |  | 0.400 | 0.694 | quasi-Poisson | Na+-transporting methylmalonyl-CoA/oxaloacetate decarboxylase, gamma subunit |
| COG3780 | -5.117 | **3E-07** |  | -4.821 | **1E-06** |  | 6.308 | **3E-10** | Poisson | DNA endonuclease related to intein-encoded endonucleases |
| COG3969 | -0.878 | 0.393 |  | 0.997 | 0.333 |  | 0.905 | 0.379 | quasi-Poisson | Predicted phosphoadenosine phosphosulfate sulfotransferase |
| COG4003 | -5.803 | **7E-09** |  | -8.189 | **3E-16** |  | 7.908 | **3E-15** | Poisson | Uncharacterized protein conserved in archaea |
| COG4046 | -6.417 | **1E-10** |  | -6.809 | **1E-11** |  | 8.155 | **4E-16** | Poisson | Uncharacterized protein conserved in archaea |
| COG4199 | -0.986 | 0.324 |  | -0.368 | 0.713 |  | 1.029 | 0.304 | Poisson | Uncharacterized protein conserved in bacteria |
| COG4316 | -4.048 | **9E-04** |  | -5.302 | **7E-05** |  | 5.228 | **8E-05** | quasi-Poisson | Uncharacterized protein conserved in bacteria |
| COG4416 | -1.538 | 0.144 |  | 0.048 | 0.963 |  | 1.797 | 0.091 | quasi-Poisson | Mu-like prophage protein Com |
| COG4640 | 5.348 | **7E-05** |  | 5.294 | **7E-05** |  | -5.845 | **2E-05** | quasi-Poisson | Predicted membrane protein |
| COG4693 | -0.536 | 0.600 |  | 1.182 | 0.255 |  | 0.482 | 0.636 | quasi-Poisson | Oxidoreductase (NAD-binding), involved in siderophore biosynthesis |
| COG4739 | 5.421 | **6E-05** |  | 4.595 | **3E-04** |  | -5.774 | **3E-05** | quasi-Poisson | Uncharacterized protein containing a ferredoxin domain |
| COG4754 | -4.351 | **1E-05** |  | -4.741 | **2E-06** |  | 5.554 | **3E-08** | Poisson | Uncharacterized conserved protein |
| COG4808 | -0.591 | 0.563 |  | 1.213 | 0.243 |  | 0.573 | 0.575 | quasi-Poisson | Uncharacterized protein conserved in bacteria |
| COG4810 | -0.643 | 0.529 |  | 0.996 | 0.334 |  | 0.666 | 0.515 | quasi-Poisson | Ethanolamine utilization protein |
| COG4812 | -0.804 | 0.433 |  | 0.760 | 0.458 |  | 0.887 | 0.388 | quasi-Poisson | Ethanolamine utilization cobalamin adenosyltransferase |
| COG4881 | -4.004 | **6E-05** |  | -4.326 | **2E-05** |  | 5.168 | **2E-07** | Poisson | Predicted membrane protein |
| COG4888 | -4.300 | **2E-05** |  | -4.782 | **2E-06** |  | 5.512 | **4E-08** | Poisson | Uncharacterized Zn ribbon-containing protein |
| COG4893 | -2.000 | 0.063 |  | -0.573 | 0.574 |  | 2.466 | **0.025** | quasi-Poisson | Uncharacterized protein conserved in bacteria |
| COG4900 | -4.300 | **2E-05** |  | -4.782 | **2E-06** |  | 5.512 | **4E-08** | Poisson | Predicted metallopeptidase |
| COG4904 | -4.300 | **2E-05** |  | -4.782 | **2E-06** |  | 5.512 | **4E-08** | Poisson | Uncharacterized protein conserved in archaea |
| COG4909 | -1.411 | 0.177 |  | 0.411 | 0.687 |  | 1.555 | 0.139 | quasi-Poisson | Propanediol dehydratase, large subunit |
| COG4910 | -1.411 | 0.177 |  | 0.411 | 0.687 |  | 1.555 | 0.139 | quasi-Poisson | Propanediol dehydratase, small subunit |
| COG4917 | -0.559 | 0.584 |  | 1.109 | 0.284 |  | 0.552 | 0.589 | quasi-Poisson | Ethanolamine utilization protein |
| COG4919 | -4.300 | **2E-05** |  | -4.782 | **2E-06** |  | 5.512 | **4E-08** | Poisson | Ribosomal protein S30 |
| COG5043 | -0.021 | 0.983 |  | 1.421 | 0.155 |  | -1.044 | 0.296 | Poisson | Vacuolar protein sorting-associated protein |
| COG5061 | -4.388 | **1E-05** |  | -7.734 | **1E-14** |  | 6.738 | **2E-11** | Poisson | Oxidoreductin, endoplasmic reticulum membrane-associated protein involved in disulfide bond formation |
| COG5096 | 2.572 | **0.020** |  | 2.115 | 0.051 |  | -3.016 | **0.008** | quasi-Poisson | Vesicle coat complex, various subunits |
| COG5125 | 3.314 | **9E-04** |  | 2.454 | **0.014** |  | -3.262 | **0.001** | Poisson | Uncharacterized conserved protein |
| COG5131 | -4.300 | **2E-05** |  | -4.782 | **2E-06** |  | 5.512 | **4E-08** | Poisson | Ubiquitin-like protein |
| COG5137 | -6.249 | **4E-10** |  | -7.317 | **3E-13** |  | 8.369 | **<2E-16** | Poisson | Histone chaperone involved in gene silencing |
| COG5146 | 3.019 | **0.008** |  | 3.115 | **0.007** |  | -3.490 | **0.003** | quasi-Poisson | Pantothenate kinase, acetyl-CoA regulated |
| COG5148 | -3.681 | **2E-04** |  | -7.015 | **2E-12** |  | 5.682 | **1E-08** | Poisson | 26S proteasome regulatory complex, subunit RPN10/PSMD4 |
| COG5157 | -1.307 | 0.191 |  | -5.889 | **4E-09** |  | 2.278 | **0.023** | Poisson | RNA polymerase II accessory factor |
| COG5183 | 2.961 | **0.009** |  | 2.619 | **0.019** |  | -3.407 | **0.004** | quasi-Poisson | Protein involved in mRNA turnover and stability |
| COG5222 | -6.150 | **8E-10** |  | -9.064 | **<2E-16** |  | 9.336 | **<2E-16** | Poisson | Uncharacterized conserved protein, contains RING Zn-finger |
| COG5232 | -4.087 | **4E-05** |  | -2.475 | **0.013** |  | 4.759 | **2E-06** | Poisson | Preprotein translocase subunit Sec62 |
| COG5243 | -12.070 | **<2E-16** |  | -13.530 | **<2E-16** |  | 15.750 | **<2E-16** | Poisson | HRD ubiquitin ligase complex, ER membrane component |
| COG5245 | -0.732 | 0.475 |  | 0.968 | 0.347 |  | 0.742 | 0.469 | quasi-Poisson | Dynein, heavy chain |
| COG5260 | -5.068 | **4E-07** |  | -4.700 | **3E-06** |  | 6.216 | **5E-10** | Poisson | DNA polymerase sigma |
| COG5322 | 2.866 | **0.011** |  | 3.358 | **0.004** |  | -3.560 | **0.003** | quasi-Poisson | Predicted dehydrogenase |
| COG5411 | -3.532 | **4E-04** |  | -3.807 | **1E-04** |  | 3.514 | **4E-04** | Poisson | Phosphatidylinositol 5-phosphate phosphatase |
| COG5427 | 2.016 | 0.061 |  | 1.468 | 0.161 |  | -2.359 | **0.031** | quasi-Poisson | Uncharacterized membrane protein |
| COG5491 | -5.776 | **8E-09** |  | -4.837 | **1E-06** |  | 7.020 | **2E-12** | Poisson | Conserved protein implicated in secretion |
| COG5540 | -4.300 | **2E-05** |  | -4.782 | **2E-06** |  | 5.512 | **4E-08** | Poisson | RING-finger-containing ubiquitin ligase |
| COG5545 | 4.214 | **7E-04** |  | 4.049 | **9E-04** |  | -4.803 | **2E-04** | quasi-Poisson | Predicted P-loop ATPase and inactivated derivatives |
| COG5561 | 3.846 | **0.001** |  | 3.401 | **0.004** |  | -4.285 | **6E-04** | quasi-Poisson | Predicted metal-binding protein |
| COG5574 | -1.307 | 0.191 |  | -5.889 | **4E-09** |  | 2.278 | **0.023** | Poisson | RING-finger-containing E3 ubiquitin ligase |
| COG5618 | -2.089 | 0.053 |  | -0.061 | 0.952 |  | 2.284 | **0.036** | quasi-Poisson | Predicted periplasmic lipoprotein |
| COG5625 | -4.300 | **2E-05** |  | -4.782 | **2E-06** |  | 5.512 | **4E-08** | Poisson | Predicted transcription regulator containing HTH domain |
| COG5651 | -3.434 | **0.003** |  | -4.187 | **7E-04** |  | 4.567 | **3E-04** | quasi-Poisson | PPE-repeat proteins |
| COG5656 | -1.615 | 0.126 |  | -0.050 | 0.960 |  | 1.916 | 0.073 | quasi-Poisson | Importin, protein involved in nuclear import |
| **KEGG Pathway** |  |  |  |  |  |  |  |  |  |  |
| map00563 | -2.374 | **0.018** |  | 0.259 | 0.796 |  | 2.977 | **0.003** | Poisson | Glycosylphosphatidylinositol (GPI) anchor biosynthesis |
| map04640 | -10 | **<2E-16** |  | -12.53 | **<2E-16** |  | 13.19 | **<2E-16** | Poisson | Hematopoietic cell lineage |
